# Complexity in pediatric primary care

**DOI:** 10.1017/S146342361800035X

**Published:** 2018-05-22

**Authors:** Arwa Nasir, Laeth Nasir, Ariel Tarrell, David Finken, Amy Lacroix, Swetha Pinninti, Sheryl Pitner, Molly McCarthy

**Affiliations:** 1 Department of Pediatrics, 982167 Nebraska Medical Center, University of Nebraska Medical Center, Omaha, NE, USA; 2 Creighton University School of Medicine, Omaha, NE, USA; 3 College of Public Health, 984365 Nebraska Medical Center, University of Nebraska Medical Center, Omaha, NE, USA

**Keywords:** chronic medical complexity, multimorbidity, pediatric primary care, psychosocial morbidity

## Abstract

**Background:**

The management of patients in primary care is often complicated by the presence of multiple chronic conditions and psychosocial issues that increase the complexity of the encounter and have important impacts on care. There is a paucity of literature on this subject in the pediatric population.

**Objectives:**

The aim of this study was to quantify the burden of chronic conditions in pediatric primary care.

**Methods:**

The problem lists of 3995 randomly selected patients from a community pediatric clinic and an academic hospital-based pediatric clinic in the same metropolitan area were analyzed for the presence and number of any chronic condition.

**Results:**

In total, 53% of patients suffered from at least one chronic problem, 25% had two or more chronic conditions and 5.1% had four or more conditions. Compared with the community clinic, the academic clinic had significantly more children with catastrophic complex conditions (*P*<0.001). A regression analysis showed a significant positive correlation between the number of chronic medical conditions and mental health diagnoses.

**Conclusions:**

The burden of chronic disease in the pediatric primary care setting may be significantly higher than has been previously suggested. To ensure optimal quality of care, health planners should take into account the high burden of chronic illness, psychosocial issues and multimorbidity among patients in the pediatric primary care setting, as well as the higher complexity profile of patients attending academic clinics.

## What’s new

Based on a review of a large and demographically varied sample, this research documents a high burden of chronic disease and chronic medical complexity in pediatric primary care and the significant correlation between chronic medical and mental health conditions.

## Introduction

Patients with chronic health problems are among the most expensive group of patients to care for in any health system. As a group, they sustain the highest costs and highest rates of hospitalization (Moffat and Mercer, 2015).

The proportion of patients presenting to the pediatric primary care setting with chronic illness and multimorbidity, defined as the coexistence of two or more health problems is growing (Uijen and van de Lisdonk, 2008; Pefoyo *et al*., 2015). In part, this has been attributed to advances in medical and rehabilitative management, allowing children with increasingly severe conditions to survive to older ages. Other trends that may contribute include increased rates of obesity and other chronic conditions that include allergic diseases as well as behavioral and mental health conditions (Branum and Lukacs, 2009; Perou *et al*., 2013).

These factors have driven the need for delivery of increasingly complex care in the outpatient setting. Polypharmacy, coordination with specialists and social services, lack of evidence-based guidelines for treatment of concurrent conditions and difficulty in prioritizing treatments are just some of the challenges that physicians encounter in dealing with patients with chronic disease and multimorbidity. Reports suggest that generalists encountering patients with multimorbidity experience greater workloads, time pressure and feelings of frustration (Sondergaard *et al*., 2015; Foster *et al*., 2017). It is also increasingly clear that the association of chronic disease and psychosocial issues represent a major barrier to the successful management of patients in the primary care setting, and create additional burdens for providers (Hwang *et al*., 2017).

Although the awareness of the prevalence and impact of multimorbidity is increasing, few studies on the prevalence of multimorbidity in primary care populations have included patients in the pediatric age range (Schellevis *et al*., 1993; Britt *et al*., 2008; Uijen and van de Lisdonk, 2008; van Oostrom *et al*., 2012). Most studies have calculated population prevalence rates using a pre-specified list of common chronic illnesses. These lists tend to be limited and skewed toward common problems that impact adult populations, such as coronary artery disease or hypertension. In addition, previous studies have not included psychosocial problems. In pediatric practice, conditions such as homelessness or abuse are particularly important because they often represent high levels of vulnerability to harm and so require active and ongoing monitoring and management. Often, dealing with these problems and their attendant dysfunctions are as challenging as dealing with complex medical problems. Likewise, problematic family dynamics or personality issues may complicate medical management. These are often not readily amenable to individual or multidisciplinary solutions (Campo *et al*., 1999; Kellogg *et al*., 1999; Radovic *et al*., 2015).

Attempts to estimate the numbers of children with any chronic disease in the United States have been hampered by inconsistency in the populations and settings studied, methods of recruitment, data collection and definitions utilized. These studies are further limited by the use of billing information to identify clinical complexity. In primary care, billing codes or scoring systems are often used as a proxy for the time and effort expended on patient care. However, coding data and commonly used scoring or risk adjustment systems designed for single illness paradigms may not accurately capture the complexity of health delivery in this setting (Horner *et al*., 1991; Woodward *et al*., 1998; Grant *et al*., 2011; Cederna-Meko *et al*., 2016; Hwang *et al*., 2017). The variability in disease definitions and populations is reflected in the wide range of estimates of chronic disease reported in a recent meta-analysis, which reported rates of chronic disease of 0.5–44% in various studies (van der Lee *et al*., 2007). Surveys that have used stricter criteria to identify the subset of children with the most severe or catastrophic forms of chronic disease [usually defined as those requiring constant care or the use of technology to sustain life or maintain health – which we define as Catastrophic Medical Complexity (CMC)] have estimated a prevalence in the population of <1% (Stein and Silver, 1999).

The delivery of primary care is unique in that it is influenced by a very wide range of interpersonal, medical, psychosocial and systems issues that often must be taken into account when making a diagnosis, planning care coordination, providing treatment, education or arranging follow-up (Sussman *et al*., 2006). The coexistence of more than one medical condition or the co-occurrence of medical and behavioral or psychosocial conditions increases the complexity of medical decision making and/or the visit duration (Foster *et al*., 2017). With this in mind, the aim of this study was to characterize the burden of chronic disease in the pediatric primary care setting using input from front-line primary care providers. Feedback from those clinicians providing the care is important, since the complexity experienced in primary care is not fully captured by historical costs, billing data or other administrative information.

A secondary goal of the analysis was to determine if the prevalence of patients with chronic conditions differed by patient demographic characteristics or by the location of the site where care delivery occurred, in an academic versus community clinic.

## Methods

### Research design and patient population

We conducted a retrospective review of electronic medical records (EMR) of 3998 randomly selected patients seen in two general pediatrics clinics between June 2015 and May 2016. The two clinics are part of the same practice group with a common EMR (Epic). One of the clinics is an academic ambulatory pediatric clinic located on the campus of a quaternary medical center, and the other a community primary care office in a suburban location.

A computer-generated random sample of 1999 unique medical record numbers (MRN) was generated from each clinic for a total of 3998 records. Data obtained from the EMRs included age, sex, insurance status, language and the problem list of each patient. Three medical records that had no patient data associated with the MRN were excluded from the analysis. The sample size was calculated to power the study to detect a significant difference in the number of patients with CMC, between the two clinic sites.

The Institutional Review Board at the University of Nebraska Medical Center reviewed and approved the study protocol.

### Chronic disease definition

A list of chronic disease categories was generated by the research team based on review of the problem lists of the sample population (Table 1). Conditions were included in the chronic disease categories if they were chronic, and were judged by the group to require attention that might include treatment, care coordination, medication management and education over time. These included chronic physical, mental health and social problems that are commonly encountered in primary care practice.

**Table 1:** Chronic disease categories

Category	Example diagnoses
Organ system failure, transplant	Hepatic failure, renal failure, S/P liver or renal transplant
Chromosomal, genetic, and hereditary diseases and syndromes	Trisomy 21, Prader–Willi syndrome, Beckwith–Wiedemann syndrome, VATER syndrome, DiGeorge syndrome, Angelman syndrome, Turner syndrome, Ehlers–Danlos, Sturge–Weber syndrome, muscular dystrophy, tuberous sclerosis, phenylketonuria, Osteogenesis imperfecta, cystic fibrosis
Prematurity	Prematurity, preterm delivery
Developmental delays and intellectual disabilities	Developmental delay, speech delay, language delay, motor delay, intellectual disability, learning disability, dyslexia, articulation disorder, cognitive impairment
Mental health diagnoses	ADHD, mood disorders, anorexia nervosa, alcohol and drug abuse, social anxiety disorder, phobias, PTSD, OCD, binge-eating disorder, dysthymia, oppositional defiant disorder, high-risk sexual behavior
Chronic infections	Histoplasmosis, HIV infection, TB
Cancers, Malignancy	Acute lymphocytic leukemia, acute myeloblastic leukemia, giant cell astrocytoma, myeloproliferative disorder, history of Hodgkin’s lymphoma, Ewing’s sarcoma
Sensory impairment	Blindness, deafness
Failure to thrive/short stature/failure to gain weight	Failure to thrive, short stature, malnutrition
Birth defects and congenital anomalies	Congenital hip dysplasia, cleft lip/palate, tethered spinal cord, thyroglossal duct cyst, clubfoot, branchial cleft anomaly, micrognathia, gastroschisis
Obesity/overweight	BMI >85th percentile, overweight, obesity, morbid obesity
Psychosocial conditions	Teen pregnancy, sexual abuse, victim of bullying, child neglect, foster care status, non-accidental trauma, homelessness, sexually active at young age
Hematologic	Beta thalassemia major, alpha thalassemia, sickle cell disease
Endocrine	Precocious puberty, hypothyroidism, congenital adrenal hyperplasia, growth hormone deficiency, ACTH deficiency, polycystic ovarian syndrome
Metabolic	Diabetes mellitus type 1 and 2. Hypercholesterolemia, dyslipidemia, lead poisoning, alpha-1-antitrypsin deficiency, osteoporosis, metabolic syndrome
Rheumatologic	Ankylosing spondylitis, juvenile idiopathic arthritis
Neurologic	Migraines, chronic pain, periventricular leukomalacia, central sleep apnea, tic disorder, neuralgias and neuritis, hydrocephalus, seizure disorder, epilepsy, myoclonic seizure disorder, temporal lobe epilepsy, cerebral palsy, static encephalopathy
Orthopedic	Degenerative disc disease, patellofemoral disorder, Sever’s apophysitis, Legg–Calvé–Perthes disease, spinal deformity
ENT	Chronic sinusitis, cholesteatoma
GI/liver	Constipation, gastroesophageal reflux disease, eosinophilic esophagitis, Crohn’s disease, celiac disease non-alcoholic steatohepatitis
Cardiovascular	Aortic stenosis, hypertrophic cardiomyopathy, hypertension, ventricular septal defect, congenital heart disease, mitral valve regurgitation, transposition of great arteries
Renal	Polycystic kidney disease, hydronephrosis, vesicoureteric reflux, horseshoe kidney, renal insufficiency
Pulmonary	Bronchopulmonary dysplasia, obstructive sleep apnea, pulmonary nodules, chronic lung disease, respiratory insufficiency, sarcoidosis
Immunologic	Allergic rhinitis, allergy to nuts/food/pollen, IgA deficiency, immunodeficiency, environmental allergies, history of splenectomy
Dermatologic	Eczema, psoriasis, vitiligo, atypical nevi, atopic dermatitis, Kyrle’s disease
Autism	Autism, autism spectrum disorder, pervasive developmental disorder, Asperger’s syndrome
Asthma	Asthma: mild intermittent, mild persistent, moderate persistent, severe persistent, cough variant asthma, exercise-induced asthma
Technology dependence/assistance	Cardiac pacemaker, CSF shunt, CPAP, O_2_ dependence, ileostomy status, ventilator dependence, feeding by G-tube, wheelchair dependence, tracheostomy dependence, cochlear implants

ADHD=attention-deficit/hyperactivity disorder; PTSD=Posttraumatic stress disorder; OCD=obsessive compulsive disorder; HIV=human immunodeficiency virus; TB=tuberculosis; BMI=body mass index; ACTH=adrenocorticotropic hormone; ENT=ear nose and throat; GI=gastrointestinal; IgA=immunoglobulin A; CSF=cerebrospinal fluid; CPAP=continuous positive airway pressure.

Diagnostic categories were then assigned to each diagnosis on the problem list of the sample data. The initial category assignment was independently performed by two of the investigators, then compared with ensure consistency. The final categorization of the entire data set was then reviewed by the primary investigator for consistency.

Additionally, the review identified patients who met criteria for CMC defined as children with one or more conditions resulting in severe disability, needs for intensive home care, chronic disease management and specialty care, and in most cases, reliance on medical technology for survival. As with the diagnostic categories above, designation as severe medical complexity was also performed by two of the investigators independently; the results were subsequently compared for consistency, then reviewed and confirmed by the group.

### Statistical analysis

Descriptive statistics were used to characterize the demographic variables of the sample population. Bivariate analyses were used to compare the patients’ demographic information at each of the two clinic locations. Next, distribution of the number of complex conditions was examined using *χ* ^2^ tests for categorical variables and comparing medians and interquartile ranges for numeric variables. Finally, a multinomial logistic regression was conducted, which modeled the relationship between the number of chronic medical problems and the number of psychosocial/behavioral problems, while controlling for the effect of age, sex, insurance status, primary language and clinic location.

In this analysis, the outcome variable was the number of chronic complicating medical problems (none, one to two, three to five or more). The model covariates included the presence of a psychosocial or behavioral diagnosis (any versus none), age group (0, 1–2, 3–5, 6–8, 9–11, 12–15, 16+ years), sex, insurance (private, public, uninsured), language (English, Spanish, other) and clinic location (academic versus community). Data analysis was conducted in Stata 14.2.

## Results

A total of 3995 pediatric patients EMRs were included in this analysis. Demographic characteristics of patients are presented in Table 2. Patients from the academic clinic were more likely to be younger, less likely to identify English as the primary language of the family (*P*<0.001) and more likely to have public or no insurance (*P*<0.001). Patients with public insurance had a higher rate of having one or more or chronic medical problems relative risk ratio (RRR): 1.41 (1.17, 1.70) and 1.78 (1.26, 2.53), respectively. Similarly, being uninsured conferred higher risk of having one or two chronic medical problems RRR: 1.95 (1.12, 3.40).

**Table 2:** Participant characteristics

Demographic characteristics	Total (*n*=3995) *n* (%)	Academic clinic (*n*=1996) *n* (%)	Community clinic (*n*=1999) *n* (%)	*P*-value
Age (years)				
<1	401 (10.0)	248 (12.4)	153 (7.7)	<0.001
1–2	627 (15.7)	379 (19.0)	248 (12.4)	<0.001
3–5	749 (18.8)	391 (19.6)	358 (17.9)	ns
6–8	564 (14.1)	257 (12.9)	307 (15.4)	0.023
9–11	519 (13.0)	236 (11.8)	283 (14.2)	0.024
12–15	698 (17.5)	298 (14.9)	400 (20.0)	<0.001
16–19	437 (10.9)	187 (9.4)	250 (12.5)	0.002
Gender				ns
Female	1960 (49.1)	963 (48.3)	997 (49.9)	
Male	2035 (50.9)	1033 (51.8)	1002 (50.1)	
Language				
English	3632 (91.0)	1659 (83.1)	1973 (98.7)	<0.001
Spanish	294 (7.4)	286 (14.3)	8 (0.4)	<0.001
Other	67 (1.7)	51 (2.6)	16 (0.8)	<0.001
Insurance status				
Private/Tri-Care	2330 (58.3)	508 (25.5)	1822 (91.1)	<0.001
Public	1604 (40.2)	1432 (71.7)	172 (8.6)	<0.001
Uninsured	61 (1.5)	56 (2.8)	5 (0.3)	<0.001

At the bivariate level, there was a significant relationship between insurance status and the number of chronic medical problems (*χ* ^2^=17.05, *P*=0.009). Privately insured patients were less likely to have any chronic medical condition than patients who were either uninsured or publicly insured (*χ* ^2^=11.218, *P*=0.001). Patients who were publicly insured were more likely to have one or more chronic complicating medical conditions than patients with private insurance or no insurance (*χ* ^2^=9.74, *P*=0.002). There was no relationship between being uninsured and the presence of any chronic complicating medical problems. Male patients were more likely to have 1–2 and 3–5 chronic conditions [1.22 and 1.30 times, respectively, (1.07, 1.40) and (1.01, 1.68), respectively). Finally, older patients tended to have significantly more chronic conditions than younger ones.

The distribution of the number and types of chronic conditions are presented in Table 3. More than half (52.6%) of patients in the total sample had at least one chronic condition. In total, 25% had two or more chronic conditions. The proportion of children who had 1–4 chronic conditions was similar in the academic and community clinics. There was a significantly higher proportion of patients with five or more chronic conditions at the academic clinic compared with the community clinic (*P*=0.013). The academic clinic also has significantly more patients who had CMC than the community clinic (*P*<0.001).

**Table 3:** Distribution of the number of chronic conditions

	Total sample (*n*=3995) *n* (%)	Academic clinic (*n*=1996) *n* (%)	Community clinic (*n*=1999) *n* (%)	*P*-value
Chronic conditions				
No chronic conditions	1891 (47.3)	941 (47.1)	950 (47.5)	ns
Any chronic condition	2104 (52.7)	1055 (52.8)	1049 (52.5)	ns
Catastrophic Medical Complexity	124 (3.1)	78 (3.9)	46 (2.3)	<0.001
Number of chronic conditions				
0	1891 (47.3)	941 (47.1)	950 (47.5)	ns
1	1142 (28.6)	558 (28.0)	584 (29.2)	ns
2	507 (12.7)	256 (12.8)	251 (12.6)	ns
3	250 (6.3)	122 (6.1)	128 (6.4)	ns
4	91 (2.3)	49 (2.5)	42 (2.1)	ns
5+	114 (2.9)	70 (3.5)	44 (2.2)	0.024

### Chronic conditions

The frequencies of chronic conditions are shown in Table 4. Conditions identified in Table 1 with a frequency of <2% were classified under the category of ‘Other’ in Table 4.

**Table 4:** Distribution and frequency of chronic condition types by clinic location

Category	Total *n* (%)	Academic clinic *n* (%)	Community clinic *n* (%)	*P*-value
Obesity/overweight	538 (13.5)	230 (11.5)	308 (15.4)	<0.001
Allergic/immunologic	504 (12.6)	250 (12.5)	254 (12.7)	ns
Asthma	494 (12.4)	311 (15.6)	183 (9.2)	<0.001
Behavioral/psychosocial	490 (12.3)	227 (11.4)	263 (13.2)	ns
ADD/ADHD	233 (5.8)	78 (3.9)	155 (7.8)	<0.001
Depression	78 (2.0)	39 (2.0)	39 (2.0)	ns
Anxiety	103 (2.6)	34 (1.7)	69 (3.5)	<0.001
Substance use	14 (0.4)	6 (0.3)	8 (0.4)	ns
Autism	24 (0.6)	11 (0.6)	13 (0.7)	ns
Psychosocial conditions	95 (2.4)	77 (3.9)	18 (0.9)	<0.001
Other	68 (1.7)	32 (1.6)	36 (1.8)	ns
Dermatologic	377 (9.4)	231 (11.6)	146 (7.3)	<0.001
GI/liver	300 (7.5)	151 (7.6)	149 (7.5)	ns
Developmental delays and intellectual disabilities	178 (4.5)	88 (4.4)	90 (4.5)	ns
Prematurity and other perinatal complications	146 (3.7)	91 (4.6)	55 (2.8)	0.002
Neurologic	132 (3.3)	65 (3.3)	67 (3.4)	ns
Other	684 (17.1)	366 (18.3)	318 (15.9)	ns

ADD=attention deficit disorder; ADHD=Attention-deficit/hyperactivity disorder. Categories are not mutually exclusive and do not sum to 100%.

The most frequent single diagnosis was obesity, at 13.5% of the total sample and slightly more frequent in the community clinic population (Table 4).

Asthma was the second most common single diagnosis at (12.4% of the total sample). While this average is higher than the most recent national asthma prevalence of 9.5% in children, this is likely due to the higher prevalence of this illness in the academic clinic population (15.6%). Allergic rhinitis and eczema were reported in 10.7% and 9.5% of cases, respectively. Eczema was significantly more prevalent in the academic clinic population. However, food allergies were reported three times more frequently in the community clinic population.

There was no difference in the prevalence of developmental delay or autism between the two locations. The prevalence of autism was slightly lower in our sample (0.6%) than national (1.5%) and state (0.9%) estimates.

Overall, 3.1% of the total sample met criteria for CMC. The academic clinic had significantly more patients classified as CMC (3.9%) than the community clinic (2.3%) (*P*<0.001). These numbers are higher than previous community estimates of <1%.

### Behavioral and mental health diagnoses

Mental health problems were on the problem list of 12.3% patients in the total sample. Attention-deficit/hyperactivity disorder (ADHD) was the most common behavioral diagnosis. ADHD and anxiety were diagnosed twice as commonly in the community clinic population.

### Chronic disease and psychiatric comorbidity

**Figure 1:**
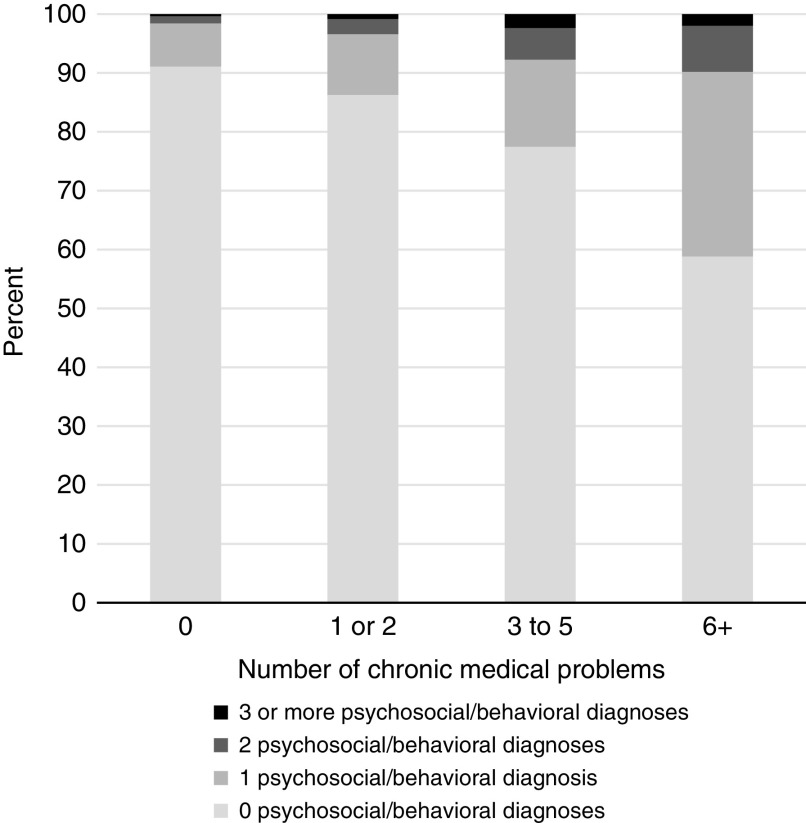
Relationship between chronic medical and psychosocial/behavioral diagnoses

There was a positive correlation between the number of chronic medical conditions and the number of psychiatric conditions (Figure 1). Patients with one psychosocial or behavioral diagnosis were 1.38 times more likely to have one or two medical problems, holding all other model covariates constant (95% CI: 1.11, 1.72). Similarly, having any psychosocial or behavioral diagnosis increased the likelihood of having three to five chronic complicating medical problems by 2.15 times (95% CI: 1.55, 2.98). Likewise, the presence of any psychosocial or behavioral diagnosis is associated with 5.83 times the risk of having six or more chronic complicating medical problems, holding all other model covariates constant (95% CI: 2.93, 11.57).

## DISCUSSION

We found that the majority (52.6%) of children in our primary care sample had a chronic condition, and that one in four patients met the definition of multimorbidity. Previous studies of outpatients that included subjects in pediatric age groups have reported rates of chronic illness that varied between 10% and 28% (Schellevis *et al*., 1993; van den Akker *et al*., 1998; Uijen and van de Lisdonk, 2008; Britt *et al*., 2008; van Oostrom *et al*., 2012; Brett *et al*., 2013). However, by consulting with primary care pediatricians to expand the diagnoses included in the definition of chronic conditions, this study provides a broader perspective on the diversity and complexity of pediatric chronic disease.

Our results also demonstrated a link between medical and psychosocial problems. Patients with psychological or psychiatric conditions were more likely to suffer from multimorbidity, and vice versa. One previously published study in adults did not find a relationship between mental health conditions and multimorbidity (Fortin *et al*., 2012), while another, larger study that included subjects in the pediatric age group reported a significant correlation (Barnett *et al*., 2012). In addition, having no insurance or public insurance predicted increased numbers of chronic health conditions among patients in our sample. This was consistent with previous research linking increased chronic disease with lower socioeconomic status and deprivation (Barnett *et al*., 2012; McLean *et al*., 2014; Violan *et al*., 2014; Pulcini *et al*., 2017).

The academic clinic served significantly more children with CMC, and more patients with greater than five comorbid chronic conditions than did the community clinic. These differences may be due to differences in ethnic, socioeconomic or environmental variables. The higher prevalence of patients with catastrophic medical conditions and comorbidity in the academic clinic could also be related to its location on the campus of a quaternary medical center in proximity to specialized services.

### Limitations of the study

In this study, we relied of data from the EMR problem list. A potential disadvantage of this strategy is that the list must be maintained by providers and kept up to date. In addition, it was generally not possible to assess the severity of medical conditions from the problem list. However, severity scales utilized to assess chronic illness in other studies such as the Cumulative Illness Rating Scale have not been validated in pediatric populations. In addition, there are data suggesting that simple disease enumeration or medication counts perform almost as well as severity scales in predicting many outcomes (Huntley *et al*., 2012).

Although the data were collected from two clinic populations that differed fundamentally in demographic characteristics, both clinics were part of the same practice plan within the same geographic boundaries of a single metropolitan area. This may limit generalizability of the data.

When developing strategies and resource allocation in care delivery redesign, it is important to ensure that the data that underpin the strategy reflect closely the clinical setting in which they will be implemented. This study differs from previous studies of multimorbidity in that it focuses exclusively on a pediatric primary care population, includes problems salient to pediatric primary care practice and relies on practicing primary care clinicians to determine which problem list diagnoses were included in the count of chronic conditions. We believe that these factors enhance the relevance and applicability of the data to the pediatric primary care setting.

## Conclusions

Chronic disease, especially multimorbidity has traditionally been considered to be unusual in the pediatric age group. In this primary care sample, we found that chronic conditions and multimorbidity were encountered commonly.

The optimal management of chronic illness and multimorbidity requires a paradigm shift in the way that the health care delivery system operates. Currently, most medical systems are designed to address single conditions. However, this often does not reflect the reality in which the primary care physician reckons with the complex interplay between multiple conditions and their effects on the overall well-being of the patient and family. Inappropriate resource allocation resulting from the underestimation of the intensity involved in caring for these populations may lead to inappropriate resource allocation. This in turn may result in undesirable outcomes that may include suboptimal management, physician burnout, unnecessary testing and referral, and family and patient dissatisfaction. Future research that further quantitates both the overall impact of chronic disease and the contribution of specific components of individual cases on the care burden may lead to more appropriate resource targeting. Better understanding the true burden of morbidity and complexity will help systems to develop innovative and effective approaches to caring for these patients. This in turn could result in improved outcomes and decreased resource use for this group of patients.

## Authors’ Contribution

A.N.: conceptualized and designed the study, directed and participated in the data analysis and interpretation, drafted the initial manuscript and the final manuscript as submitted; A.T.: participated in the data processing and analysis and critically reviewed the manuscript and approved the final manuscript; L.N.: participated in the study design and conceptualization and data analysis, participated in the drafting of the manuscript, reviewed and approved the manuscript as submitted; D.F., A.L., S. Pitner and S. Pinninti: participated in the data analysis and critical review and revision of the manuscript and approved the manuscript as submitted; M.M.: coordinated the data retrieval and management, participated in the data analysis and reviewed and approved the manuscript as submitted.

## Financial Support

Funding for this research was provided by the Department of Pediatrics, University of Nebraska Medical Center.

## Conflicts of Interest

The authors have no conflicts of interest relevant to this article to disclose.
